# Excipients in Pharmaceutical Additive Manufacturing: A Comprehensive Exploration of Polymeric Material Selection for Enhanced 3D Printing

**DOI:** 10.3390/pharmaceutics16030317

**Published:** 2024-02-24

**Authors:** Christian Muehlenfeld, Patrick Duffy, Fengyuan Yang, David Zermeño Pérez, Firas El-Saleh, Thomas Durig

**Affiliations:** 1 Ashland Life Sciences R&D, 40599 Düsseldorf, Germany; 2 Ashland Life Sciences R&D, N91 F6PD Mullingar, Ireland; 3 Ashland Life Sciences R&D, Wilmington, DE 19808, USA

**Keywords:** 3D printing, critical material properties, polymers, cellulosics, vinylpyrrolidone, bioresorbable polymers, printability, feedability, drug product

## Abstract

This review provides a comprehensive overview of additive manufacturing (AM) or 3D-printing (3DP) applications in the pharmaceutical industry, with a particular focus on the critical role of polymer selection. By providing insights into how material properties influence the 3DP process and the quality of the final product, this review aims to contribute to a better understanding of the interplay between polymers and pharmaceutical 3DP. As 3DP technologies are increasingly integrated into pharmaceutical sciences, this review contributes insights into the nuanced process of polymer selection, serving mainly as a foundational guide for researchers and formulators new to the subject seeking to harness the full potential of pharmaceutical 3DP by understanding the physicochemical properties, roles, and functions of used polymers in 3D-printed dosage forms and medical devices.

## 1. Introduction

In recent years, additive manufacturing (AM), popularly known as three-dimensional printing (3DP), has been widely applied to the fabrication of prototypes through to functional parts in various manufacturing areas, including the pharmaceutical industry. The term “3D printing” technically refers to a broad collection of additive technologies based on the deposition of materials, like creating objects from the bottom up or layer by layer. Despite the technical differentiation between the terms, “3D printing” has become the common term, and thus, 3DP and AM are used interchangeably in this review to refer to the collection of AM technologies that will be discussed.

Although there is a growing demand for technologies that enable pharmaceutical manufacturers to offer personalized and customized treatments, the uptake and utilization of 3D printing in healthcare applications is relatively new. To date, this has predominantly involved the printing of plastics and metals, such as surgical planning, prosthetics, or reconstructive surgery, and has been used to create tailored bone inserts for use in complex reconstructive surgery [1,2,3]. On the contrary, the 3D printing of pharmaceuticals is less advanced. For example, in 2015, the US regulatory agency the Food and Drug Administration (FDA) approved the first 3D-printed pill, Spritam^®^, from Aprecia Pharmaceuticals (Blue Ash, OH, USA) [4]. The applied AM process enabled the manufacturers to produce orally disintegrating tablets with a higher drug load, compared to conventional levetiracetam solid-dosage forms, and a short disintegration time due to the porous structure of the printed tablets. Like any other solid oral dosage form, the FDA affirmed that the 3D-printed tablet must comply with existing manufacturing and control regulations such as 21 CFR 200 and 21 CFR 300 [5], and they are approved according to existing regulatory and quality frameworks [6]. In 2016, the FDA released new draft guidance on the Technical Considerations for Additive Manufactured Devices to advise manufacturers who are producing devices through 3D-printing techniques. With the official document issued in 2017, the guidance provides manufacturers with recommendations for two major areas of medical device development: device design and manufacturing, and device testing [7]. Specifically for device testing, which includes device characterization, validation, and verification, the FDA recognized the unique challenges associated with AM. This starts with the impact of the 3D printer and post-printing processes on the final device performance; however, it also includes the importance of material control and the need for a robust process validation and acceptance protocol. Since then, there has been an increased interest in 3DP technologies across the pharmaceutical industry.

The main advantages associated with the use of AM technologies in the pharmaceutical area include customization, i.e., the development of personalized medications such as individualized printed dosage forms and implants [8]. Furthermore, AM technologies can contribute to bringing new therapies to the market via small-scale production in a brief time [9]. This is beneficial for the development of new drugs and therapies, especially when utilized for parenteral applications. Finally, AM helps to advance the personalization of medicines by enabling fabrication according to the patient’s treatment and dosage needs, e.g., for geriatric and pediatric patient populations who lack suitable available formulations [10], and blind or visually impaired individuals [11].

During the development of AM technologies, various definitions and terms were used that referenced specific application areas and even trademarks. The standard ISO/ASTM 52900:2021 [12] was created as a comprehensive framework to facilitate and widen communication and innovation between people involved in AM technologies. Consequently, it plays an important role in the pharmaceutical/medical sector by providing a standardized approach. According to ISO/ASTM 52900:2021, there are seven different process categories in additive manufacturing [12]. These are:

1. Binder jetting;
2. Material jetting;
3. Powder-bed fusion;
4. Material extrusion;
5. VAT photopolymerization;
6. Sheet lamination;
7. Directed energy deposition.

The first five AM technologies from the list above have been used for pharmaceutical applications. Detailed overviews and descriptions referring to these AM technologies are widely available, and thus, dedicated reviews are recommended to avoid replicating that information [13,14,15,16,17,18,19]. The same applies to characterization techniques, ranging from polymer screening, process monitoring, and optimization to intermediate and final product attributes of the printed drug products. Consequently, the reader is referred to available reviews [20]. Rather, this review should provide some guidance and direction on how to choose polymeric excipients based on their physicochemical material attributes and their influence on printability. Nevertheless, selecting a polymer with suitable feedability, printability, and product characteristics is not an easy task and requires a good understanding of how the characteristic properties of utilized polymeric excipients influence the printing process, and ultimately, final product quality.

Polymers (alone or together with other pharmaceutical excipients) play an important role in the manufacturing of conventional or advanced drug-delivery systems. In conventional manufacturing technologies, they are generally used as binders, fillers, lubricants, and solubility enhancers in oral solid dosage forms such as tablets and capsules, as well as emulsifying, suspending, or stabilizing agents and rheology enhancers in liquid and semisolid preparations. Polymers are macromolecules consisting of repeating units (or monomers) throughout their chains [21]. When two, three, or four monomer units are attached to each other, the product is known as a dimer, trimer, or tetramer, respectively. Products containing more than 200 monomer units are simply called polymers.

Polymers can be classified in different ways [21] based on the following:

- Their origin (naturally occurring, semi-synthetically modified, or synthetically manufactured), e.g., while cellulose is the basic structural component of plant cell walls and thus the most abundant of all naturally occurring polymers, cellulose ethers such as hydroxypropyl cellulose (HPC) or hydroxyethyl cellulose (HEC) are semi-synthetic polymers that become water-soluble due to the introduction of their side chains. On the other hand, polymers such as polyvinylpyrrolidone (PVP) and copovidone (PVPVA) are synthetic polymers obtained by the radical polymerization of their monomers.
- Types of monomer(s) (homopolymer or copolymer), e.g., PVP is obtained from the polymerization of N-vinyl-pyrrolidone (NVP) solely, while PVPVA is a copolymer obtained from the polymerization of NVP with vinyl acetate.
- Gross topology of the chain structure (linear, branched, or crosslinked), e.g., whether the polymer chain or chain assembly can be mapped onto a one- (linear), two- (branched), or three- (crosslinked network) dimensional object.
- Interaction with water (water-soluble or water-insoluble), e.g., hydroxypropyl cellulose (HPC) is a water-soluble cellulose ether, while ethyl cellulose (EC) does not dissolve in water.

Since all these properties will impact the final properties of the material [22], the right selection of polymers is crucial to facilitate the processing of the active pharmaceutical ingredient (API) into a dosage form, e.g., by providing the required physical stability to incorporated APIs or to modulate the drug dissolution and release. Thus, it is important to understand which polymeric excipients are suitable for which 3D-printing process, since polymer selection based on printing properties is key for the successful printing of the final drug product. Furthermore, despite being a processing aid in the printing process, the material choice will impact the mechanical properties and drug release and, thus, the overall performance of the final printed product.

This review is structured into three parts that should help readers in understanding the complexities of polymer printability in AM:

- Explanation of the significance of material properties in successful 3D printing.
- Introduction to the key process stages of 3D printing—feeding, deposition and adhesion—and a discussion about the importance of these key determinants of printability.
- Discussion of pharmaceutical polymers (cellulose ethers, polyvinyl polymers, and bioresorbable polymers) and their material properties (solubility, viscosity, rheology, and mechanical characteristics) for their use in different AM technologies.

The selection and properties of materials are directly linked to the AM process, and the working principle of each technique defines the required properties of the material to be used. Table 1 lists the commonly used polymers for pharmaceutical AM applications. While some polymers such as ethyl cellulose, hydroxypropyl methylcellulose, and povidone (PVP) are added to the printing ink due to their rheological properties or impact on the ink’s surface tension [23], other polymers may cause nozzle clogging when added to the printing ink and, therefore, might be preferably included in the solid substrate [24]. The same polymers are used in PAM extrusion technologies; however, in this case, they are added to help wet the dry solid substrate, thus contributing to interlayer adhesion, and consequently, they support the mechanical structure of the final printed product [25]. In recent years, thermoplastic polymers such as copovidone and povidone, as well as cellulosics such as ethyl cellulose and hydroxypropyl cellulose, have been extensively used in spray-drying and hot-melt extrusion applications to solubilize poorly soluble active pharmaceutical ingredients (APIs). In addition to solubilization, the same polymers also help stabilize the metastable amorphous form of the API, leading to the formation of amorphous solid dispersions [26]. Accordingly, these polymers have been used in melt-based AM technologies such as fused deposition modeling (FDM) as well; however, not all the thermoplastic polymers traditionally used in hot-melt extrusion (HME) perform well in FDM. The necessary higher processing temperatures in FDM compared to HME, as well as the requirements to show immediate drug release, might limit the use of some HME polymers in FDM [27]. Table 1 provides a summary of the commonly used polymers utilized in pharmaceutical-relevant 3D-printing applications. Publications selected for this review had to fulfill the following criteria:

- The additive manufacturing technology was clearly described and fit into the five ISO/ASTM 52900:2021 pharmaceutically relevant categories listed above.
- The polymers described were relevant for the functionality of the printed drug product and the authors provided sufficient information on the polymer types and grades used.
- The scope of the work was pharmaceutical or biomedical research with a particular focus on processability.
- There was no restriction on the publication period for manuscripts; however, more focus was put on papers published within the last 5 years.

## 2. Printability of Polymers—Important Material Characteristics

**Figure 1:**
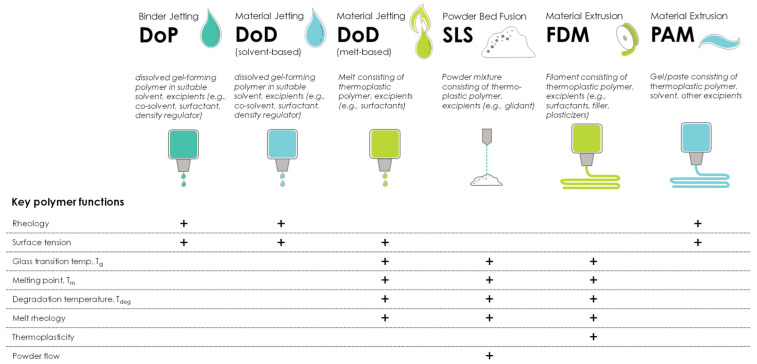
Classification of pharmaceutically relevant AM technologies and examples of the key polymer material properties affecting printability; modified according to [20]. The “+” indicates that the specific polymer function is important for the AM technology listed in that column of the table. DoP = drop-on-powder printing; DoD = drop-on-demand printing; SLS = selective laser sintering; FDM = fused deposition modeling; PAM = pressure-assisted micro-syringe.

Each AM technology requires materials with certain physicochemical properties for successful printing, and it is important to select polymers that enable successful printing in the desired AM manufacturing technology. In order to allow for a systematic overview of material requirements for different AM technologies, the 3DP process is divided into three printing process stages: feeding, deposition, and adhesion (to the previously printed layer, the build-plate of the printer, or another substrate). These three process stages have been identified as key determinants of product quality in most 3DP technologies [20], and material requirements and key polymer functions will be different for each process stage. Figure 1 summarizes the key polymer functions for printability in 3DP processes, according to Govender et al. [20].

The different 3DP technologies require feedstocks with varying material properties. The feedability of the feedstock into the printer is a decisive starting point for successful 3D printing in most cases. This is only a non-mandatory requirement for VAT photopolymerization and powder-bed fusion methods, as these processes lack nozzle-based working principles requiring the feedability of the feedstock. Powder-bed fusion methods such as SLS produce objects from powder materials using a thermal energy source to melt the surface particles layer by layer. This process requires a powder blend consisting of thermoplastic polymers as a feedstock with sufficient powder flow properties [107]. In VAT photopolymerization techniques, liquid resins are selectively crosslinked by light irradiation. This process requires photosensitive resins, typically composed of methacrylates or acrylic esters, and a photoinitiator for pharmaceutical applications [108]. Photosensitive hydrogels can be used as well. In material extrusion processes, a printable filament flows through a nozzle and is deposited on a construction platform. In material extrusion using FDM, a filament serves as the feedstock and is fed through a heated nozzle, acting as a piston to push itself through the nozzle [109]. This process depends strongly on the mechanical properties of the filament, and thus, the polymer type and content. Consequently, common challenges in FDM are related to the stiffness and toughness of the filament: It needs to be tough enough to be conveyed and rolled up on the spool to serve as a feedstock for the printing process, but also stiff enough to push the melt through the rotating drive gears of the hot printer nozzle like a piston. Conversely, a brittle filament cannot be rolled up and collected on the spool, and more importantly, it will break due to flexion in the nozzle and under the force of the rotating drive gears, causing interruptions or even a full stop of the printing process. On the contrary, while too soft a filament will not break in the printing process, it tends to bend or deform in the printing nozzle above the heating element during feeding, causing a stop of the printing process due to the lack of a piston pushing the material through the nozzle. Consequently, thermoplastic behavior characteristics are key for processability in FDM printing. This includes material properties such as flexibility, brittleness, stiffness, toughness, melting point (T_m_), glass transition temperature (T_g_), thermal degradation temperature (T_deg_), and melt-flow index. The main difference between material extrusion using FDM and PAM printing is the physical properties of the material employed. For the PAM printing technique, semisolid materials such as gels and pastes are processed by a pneumatical or mechanically driven piston. This requires an extra processing step before feeding it into the 3D printer and involves the dispersion or dissolution of the gel-forming polymer. Material jetting processes such as DoD are based on the 2D inkjet working principle. In solvent-based DoD, a jet of solvent droplets is deposited layer by layer, and like PAM printing, an extra pre-processing step is required to disperse or dissolve the gel-forming polymer before feeding it into the printer. Melt-based DoD printing requires the feeding of a molten polymer instead of a solvent.

After the feedability of the feedstock, deposition from the nozzle is the second key determinant of printability for most AM technologies [20]. While it does not matter whether deposition occurs through a heated or a non-heated nozzle, the polymers used require different material properties depending on the deposition method. For instance, deposition through a heated nozzle (e.g., as in melt extrusion deposition or melt-based DoD printing) requires polymers to show favorable thermal and melt rheological properties for successful deposition, such as good flow through the nozzle under existing shear forces and temperatures without degradation and subsequent adhesion to the construction platform. Extrusion-based PAM printing processes require similar rheological properties to the used polymer gel or paste to ensure material flow through the nozzle without blockage under the existing shear forces [18]. This explains the importance of the rheological properties of polymers used in PAM, like HPMC, HEC, and PVP. Since these polymers typically require only small amounts to set the required viscosity, other excipients can be added to increase the solid content of the formulation, which helps to strengthen and shape the print structure, and thus, improve product performance [20]. Solvent-based material jetting processes require a specific surface tension and viscosity of the used solution for drop dynamics, and therefore, polymers may be added to the solvents to obtain the required solution properties [110]. Table 1 also lists typical polymers that have been used for material jetting techniques aiming to control both surface tension and viscosity. The same properties apply to melt-based DoD printing in general; however, if molten polymers are the primary carrier, one should consider that surface tension and viscosity are temperature-dependent material properties [111]. Table 2 highlights exemplary surface tension data for some cellulose ethers [112] measured from 0.1% (*w*/*w*) solutions. The data indicate that the chemical structure is the primary determining factor of surface tension, while there is no or only minor impact from molecular weight. While 0.1% (*w*/*w*) solutions of carboxymethylcellulose sodium yield a surface tension similar to that of water (of approximately 71 mN/m), 0.1% (*w*/*w*) solutions of HPC yield the lowest surface tension values: 41.1–41.7 mN/m. The authors explained this by the higher degree of substitution (DS) in the HPC samples [112] and the partially hydrophobic nature of the side chains. They also suggested that the comparably higher surface tension of 51–54 mN/m of HPMC results from the low degree of molar substitution (MS). Higher surface tension is hereby a result of the larger number of hydroxyl groups present on the cellulose backbone coming from the lower MS. The surface tension of hydroxyethyl cellulose is slightly lower compared to HPMC, with values ranging from 46 to 49 mN/m. The lower surface tension of HPC is due to the higher MS of HPC, which is approximately 4.0, and thus, the greater contribution of the hydroxypropyl moiety. On the contrary, HEC has a typical MS of 2.6 with a slightly less hydrophobic nature of the hydroxyethyl side chains in the polymer [112].

Powder-bed fusion SLS technology is an example of a pharmaceutical 3D-printing process that lacks deposition from the nozzle: powdered materials or mixtures are directly fed from a reservoir to the build-plate using a sled, and thus, properties such as the powder flow, particle size distribution, and particle morphology of the polymers used are of major importance [113].

Finally, after feeding and deposition, the last key determinant of printability is adhesion to the printer build-plate, to another substrate, or to the previously printed layer [20]. Polymers contribute in two ways to achieve the best adhesion in DoP and DoD material jetting applications: the rheological properties and surface tension of the printing ink can be improved or modified by adding the polymer to the ink, or the polymers are used as a substrate onto which the droplets of the printing ink are deposited. The same applies in FDM printing; however, adhesion is not only related to rheological properties and surface tension but also to the mechanical properties of the polymer [27]. The same thermal properties (melt viscosity and melt surface tension) are key to supporting powder particle coalescence in SLS applications; however, narrow particle size, appropriate particle morphology, and powder adhesion are essential for successful layer-to-layer adhesion without the formation of unwanted voids [20,114].

To summarize, different AM technologies require different critical material properties of the polymers used. Dividing the printing process into the three steps of feeding, deposition, and adhesion helps in grouping suitable materials based on the required printing process and step.

## 3. Pharmaceutical Polymers in Additive Manufacturing

### 3.1. Cellulosic Polymers

Cellulose derivatives are important excipients widely used in the pharmaceutical industry. This section provides an overview of pharmaceutically relevant cellulose ethers and their applications in AM. Cellulose is a polysaccharide of natural origin, composed of linear chains of 1-4-linked β-d-anhydroglucopyranose units of variable length, generally synthesized by plants, with wood and cotton fibers being the primary sources of cellulose for pharmaceutical applications. Due to intramolecular and intermolecular hydrogen bonds, cellulose is not soluble in water, although it is a highly hydrophilic polymer due its high degree of crystallinity and structure [115].

**Figure 2:**
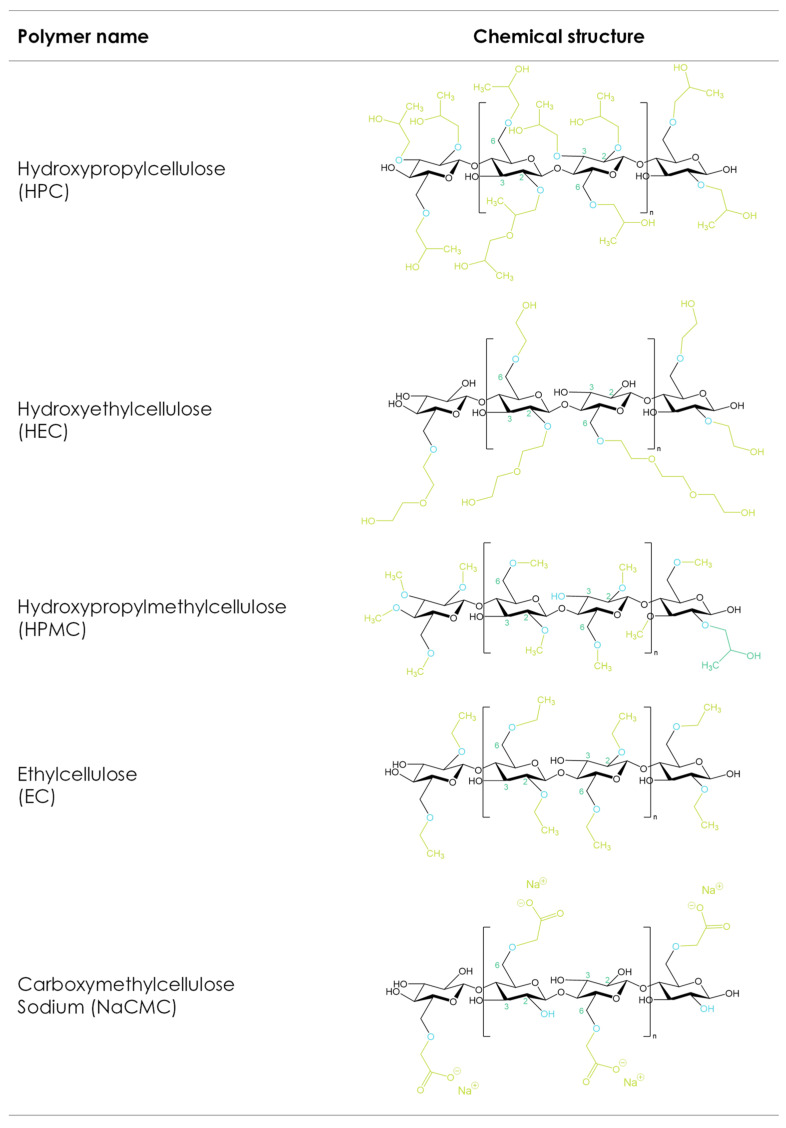
Cellulose ether derivatives.

The etherification of the hydroxyl groups in the glucose ring is a strategy that changes the cellulose’s physicochemical mechanical properties (Figure 2). Carboxymethyl cellulose sodium (CMC), hydroxyethyl cellulose (HEC), hydroxypropyl methylcellulose (HPMC), hydroxypropyl cellulose (HPC), and ethyl cellulose (EC) are the most used cellulose derivatives in pharmaceutical formulations [116,117]. The main differences between the different cellulose ethers depend on the chemistry, degrees of substitution, and distributions of the substituted groups as well as the molecular weight [118]. These properties influence solubility, viscosity in solution, surface activity, gelling performance, rheological behaviors in melt and solution, and mechanical characteristics, and thus make different cellulose ethers suitable for different 3D-printing applications.

#### 3.1.1. Carboxymethyl Cellulose Sodium (CMC)

Carboxymethyl cellulose sodium (CMC) is the sodium salt of the carboxymethyl ether of cellulose, formed by the reaction of cellulose with monochloroacetic acid. During the synthesis of CMC, the hydroxyl groups of the anhydroglucose unit are substituted by carboxymethyl groups. Modification of the molecular weight (MW) of CMC leads to a multitude of grades with variable viscosities, with the average MW ranging from approximately 49,000 to 725,000 Da [22]. Furthermore, CMC grades can be classified based on their degree of substitution (DS), which is a crucial factor in determining the physical properties of CMC, having a direct impact on material properties such as solubility, rheology, and salt tolerance. As can be seen from the structural formula in Figure 2, there are three hydroxyl groups in each anhydroglucose unit in cellulose. The number of hydroxyl groups substituted per anhydroglucose unit in any reaction is defined as the degree of substitution or DS. In the case of CMC, the maximum theoretical DS would be 3.0 if all three hydroxyls were replaced by carboxymethyl; however, this is impossible in practice. The pharmaceutically relevant grades of CMC range from DS values of 0.7 and 0.9 to 1.2. While the corresponding sodium content for DS 0.7 and 0.9 ranges from 6.5 to 9.5% (*w*/*w*), the higher DS value of 1.2 results in a higher sodium content of 10.4–12% (*w*/*w*).

In recent years, AM has been used to develop 3D-printed biocompatible structures for drug-delivery and tissue-engineering applications; however, this requires the development of new biocompatible (hydrogel) inks. CMC is a promising candidate for the preparation of hydrogels (inks) since it is a natural, biocompatible, and biodegradable polymer and has good solubility in water with multiple carboxyl groups. Among all cellulose ethers, CMC, in particular, has recently been reported as a useful structural component of bioinks for wound healing due to its matrix-forming capability, cell compatibility, and crosslinking feasibility [119]. Despite all these benefits, CMC-based inks might show insufficient mechanical stability for printed structures and, thus, the incorporation of additional additives might be necessary [120]. Diaz-Gomez et al. developed 3D-printed scaffolds for the healing of diabetic wounds utilizing CMC. Different concentrated CMC dispersions ranging from 10 to 20% (*w*/*v*) were assessed for their printability. Among those, the 15% (*w*/*v*) CMC–citric acid ink showed sufficient rheological properties and stability during storage, thus bringing the project to the next phase, which includes clinical trials [29]. Wang et al. modified CMC with glycidyl methacrylate (GMA) and ε-polylysine (CP), resulting in hydrogels with a high compression modulus (238 kPa), stable rheological properties, and effective degradability [121]. Cui et al. selected CMC to form a fine paste with good flowability, extrudability, and buildability to prepare rapid-release tablets with a smooth appearance and sufficient mechanical properties [28]. Panraksa et al. utilized a syringe extrusion 3D-printing technique to study the feasibility of 3D-printed orodispersible films (ODFs). The authors evaluated different hydrophilic film-forming polymers, including HPMC, HEC, and CMC, as printing materials. Among the formulations evaluated, 5% (*w*/*v*) CMC solutions showed good printing resolutions and accurate dimensions. Furthermore, the 3D-printed ODFs containing CMC showed disintegration within 3 s, probably related to the high wettability, roughness, and porosity on the surface of the ODF [30]. All these applications demonstrated that CMC-based hydrogels are successful facilitators in 3D printing for tissue engineering and drug-delivery systems. On the contrary, the use of CMC is limited in material jetting and FDM-based AM technologies because of the poor thermal plasticity of the polymer.

#### 3.1.2. Hydroxypropyl Methylcellulose (HPMC)

**Figure 3:**
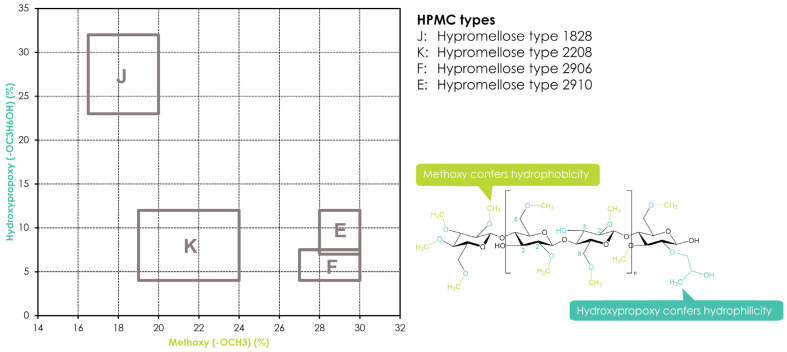
Substitution of HPMC and its different substitution types according to USP/NF and EP pharmacopeias. The rectangles in the graph depict the compendial ranges for MeO and HPO for the different HPMC types.

Hydroxypropyl methylcellulose or hypromellose (HPMC) is a cellulose ether prepared by reacting alkali cellulose in two steps: first, methyl chloride is added to introduce methoxy groups, followed by propylene oxide to introduce hydroxypropyl groups. Since the added hydroxypropyl group introduces a secondary hydroxyl group that can also be etherified during the manufacturing of HPMC, several types of HPMC are available with varying degrees of substitution (DS) and molar substitution (MS). The European pharmacopeia, USP/NF, and other pharmaceutical compendia differentiate the different substitution types of HPMC using four-digit numbers behind the non-commercial name (Figure 3), with the first two digits referring to the approximate percentage content (per weight) of the methoxy groups and the second two digits referring to the approximate percentage content of the hydroxypropoxy groups. From a commercial point of view, these different grades of HPMC were simplified by using single letters instead of four-digit numbers, i.e., “K” for HPMC 2208, “E” for HPMC 2910, and “F” for HPMC 2906. The molecular weight for all types of HPMC is in the range of 10–1500 kDa [117]. Since the viscosity of a HPMC solution corresponds to its molecular weight, the commercially available types add a suffix indicating the viscosity of a 2% aqueous solution (*w*/*w*) at 20 °C, followed by a designated “M” (multiplier of 1000) if needed, i.e., Benecel™ K100M hypromellose.

Variations in the fundamental structural properties of HPMC such as molecular weight, degree of substitution (DS), and the substitution pattern, which are due to the chemical structure of the polymer, will impact material properties such as elastic modulus, plasticity, glass transition temperature (T_g_), and thermal gelation temperature in aqueous solutions, but they will also affect swelling, diffusion, and drug-release rate in pharmaceutical dosage forms [22,122]. HPMC has been widely used to form swellable–soluble matrices in hydrophilic oral sustained-release formulations since it is a water-soluble, non-ionic cellulose derivative and its solutions are stable within the pH range of 3–11.

Furthermore, HPMC-based matrices are non-cleavable by enzymes and thus are not affected by the pH of the gastrointestinal fluids, leading to robust and reproducible drug-release profiles in vivo. Recently, HPMC has gained interest in new applications such as AM due to its thermal gelation properties: while HPMC is soluble in cold water, forming a viscous colloidal solution, it becomes less soluble in water and will build up a gel upon heating. This thermo-reversible sol–gel transition is due to hydrophobic interactions between the methoxyl groups of hypromellose during polymer dehydration at high temperatures [123,124]. Accordingly, this behavior is not only relevant for HPMC but also for other cellulose ethers containing methoxyl groups, such as hydroxypropyl cellulose. The temperature at which this effect happens is called the gelation point and is a function of the HPMC type (number of methoxy groups) and concentration (ionic strength of the solution) ranging from 50 to 90 °C. While HPMC E types (2910) have a gelation point in the range of 65 °C, necessitating higher water temperatures for solution preparation [125], HPMC types with lower numbers of methoxyl groups will show lower thermogelation, respectively. The gelation of HPMC solutions with increasing temperature comes along with a visible change from a homogeneous solution to separated aqueous and polymer phases. This effect is called the cloud point and visualizes the phase transition due to the association of the polymer with relatively large aggregates [122]. It is noteworthy to mention that the cloud point and the gelation temperature of hypromellose are not necessarily the same, and the cloudiness of the solution may be observed prior to the gelation point in many cases.

However, since the gelation process is strictly reversible and HPMC will redissolve upon cooling, this thermally induced sol–gel transition makes HPMC a suitable ingredient for 3D-printing inks, as shown by Cheng et al., who studied the impact of the rheological properties of HPMC-containing hydrogels. Using theophylline as a model drug, the authors investigated the impact of the polymer on the printability and printing quality of their fabricated tablets. Two grades of HPMCs (E4M and K4M) were evaluated at different concentrations. A concentration of 12% (*w*/*w*) HPMC K4M in the hydrogel was recognized as the best carrier to print flexible dosage combinations with theophylline due to its great extrudability and shape-retention ability. The 3D-printed HPMC tablets showed sustained drug release over 12 h via both diffusion and erosion mechanisms from the matrix [74]. Elbadawi et al. used pressure-assisted micro-syringe (PAM) 3D printing to print films containing pullulan (PUL), using HPMC as a delivery system. HPMC was found to improve the mechanical properties of the PUL films, increasing tensile strength from 8.9 to 14.5 MPa and elastic moduli up to 1.56 GPa, respectively [25].

Different from CMC and HEC, HPMC exhibits some degree of thermal plasticity when the processing temperature is above its T_g_, which is around 170–198 °C. The high T_g_ values, along with a relatively high melt viscosity, make it difficult to use HPMC without plasticizers [116]. While most available HPMC grades are unsuitable for thermal extrusion processes alone, certain grades and combinations with other thermoplastic polymers and/or plasticizers help to facilitate the extrusion process to obtain filaments with adequate mechanical properties for 3D printing. Zhang et al. combined hot-melt extrusion (HME) and FDM 3D printing using HPMC E5 and K100M together with acetaminophen (APAP) as a model drug. Filaments were manufactured by hot-melt extrusion and then transferred to the FDM printer. Solid-state characterization showed that the API was dissolved or molecularly dispersed into the polymer matrix and thus formed a solid dispersion. While the HPMC E5 was found to be a suitable filament for 3D printing with good flexibility, toughness, and stiffness, the higher-molecular-weight HPMC K100M could not be printed due to its high molecular weight and thus high melt viscosity. Both grades of HPMC required higher HME processing temperatures compared to other cellulose derivatives. Furthermore, the fabricated tablets did not result in optimal processability, indicated by rough printing paths due to the relatively high viscosity of the HPMC grades tested [52]. While the results from Zhang et al. showed that the HPMC filaments displayed high stiffness (high breaking stress) and toughness (high breaking distance), the filaments had rough surfaces. Thus, it was possible to feed the HPMC filaments into the printer, but the printing process was difficult because of the filaments’ rough surfaces and high melt viscosity during FDM printing. In order to overcome the limitations of HPMC-based filaments with respect to printability, filaments were prepared using binary polymer blends of HPMC E5 with hydroxypropyl cellulose (HPC EF or LF grades) or ethyl cellulose (EC N14 grade) and were found to perform better than single-polymer-based formulations [53]. In another study, Kadry et al. [69] investigated the effect of geometry on the drug-release profiles of FDM-printed HPMC tablets. However, it is important to emphasize that the HPMC used in this study (Affinisol™ HPMC HME 15LV) was of a non-compendial HPMC grade, with a different polymer substitution architecture compared to the allowed ranges of USP and Ph. Eur. pharmacopeias. While it showed a similar degree of methoxy substitution and a similar viscosity to the comparable regular HPMC viscosity and substitution grade, there was an increase in total substitution. The total substitution of the HPMC HME polymer grades was 47.0–59.0% yielding a T_g_ of around 90 °C [126]. This is comparable to the case of ethyl cellulose—a polymer with a typical total substitution of 48–49.5% and a T_g_ of around 133 °C. However, by successfully varying the geometric parameters, patterns, and infill densities of a drug-containing core, Kadry et al. [69] achieved different release profiles from the tablets.

#### 3.1.3. Hydroxypropyl Cellulose (HPC)

Hydroxypropyl cellulose (HPC) is cellulose ether manufactured by reacting alkali cellulose with propylene oxide at an elevated temperature and pressure. It is a highly substituted cellulose ether since the added hydroxypropyl group containing an active hydroxyl group can be further etherified during the manufacturing process, resulting in additional side-chain extension. As a result, the molar substitution (MS), which refers to the number of moles of hydroxypropyl groups per anhydroglucose ring, will be higher than the degree of substitution (DS) [22]. The MS of HPC ranges typically between 3.4 and 4.1, and thus, the hydroxypropyl substituents comprise up to 80% of the weight of HPC. The high MS results in significant changes in material properties compared to other water-soluble cellulose ethers, e.g., HPC is significantly more thermoplastic and less hygroscopic than HPMC. Furthermore, it is fully soluble in water and polar organic solvents (ethanol, methanol, isopropyl alcohol, acetone), combining several hydrophobic and hydrophilic material properties. Because of these properties, HPC is a suitable candidate for solution-based printing inks, as used in PAM printing. Sjöholm et al. fabricated thin orodispersible HPC films with a printing ink comprised of 16% (*w*/*w*) low-molecular-weight HPC EF in a solution of water and ethanol [66]. Cui et al. [67] prepared high-drug-loaded tablets (96% *w*/*w* levetiracetam) in different geometrical shapes by adding 2% of a medium-molecular-weight-grade HPC MF as a binder and 2% croscarmellose sodium as a disintegrant. The 3D-printed tablets showed acceptable ranges for tablet-breaking force, tablet friability, weight variation, and drug content. Abdella et al. fabricated estradiol-containing films using a formulation containing HPC in PAM printing. The results indicated that different infill patterns affected the film’s mechanical properties and its drug-release kinetics [75].

Commercially available grades of HPC are available in different MW grades, with values ranging from 20,000 to 1,500,000 Da (Table 3).

Within a specific viscosity grade, HPC is further available in different particle size grades. Infanger et al. [24] evaluated four different HPC qualities of two different viscosities on a drop-on-powder (DoP) printer. While the dissolution and disintegration behavior of the 3D-printed tablets mainly depended on the MW (and thus, viscosity) of the HPC grade used, the friability mainly depended on the particle size of the employed binder. Finer particle size grades resulted in less-friable tablets, and higher-MW HPC grades resulted in slower dissolution and disintegration times due to their higher viscosity and slower hydration rate. Goyanes et al. fabricated solid dispersions of itraconazole as a model drug using different grades of HPC, with MW ranging from 20 to 140 kDa. As the results of Infanger suggested, the fabricated tablets comprised of the lower-MW HPC exhibited faster drug release compared to the grades with higher MWs [62].

In addition to HPC’s good solubility in different solvents, it also shows excellent thermal plasticity and thermostability (with a degradation temperature of 227 °C [127]) due to its unique molecular structure, making it suitable for processes that require melting and extrusion, such as fused deposition modeling (FDM). In contrast to other cellulosic polymers, there is only inconsistent information on the T_g_ of HPC available, with various T_g_ values reported. Rials and Glasser [128] observed that HPC is a semicrystalline material rather than a fully amorphous one, showing a T_g_ of 19 °C and a melting point of 220 °C. Picker-Freyer and Durig [129] also concluded that HPC is a semicrystalline material, where they observed T_g_ values in the range of 0 to 5 °C in the presence of 1–10% moisture in the polymer. The marked reduction in the T_g_ as moisture increases was connected to increased plasticity with an increasing moisture content. Meena et al. [127] reported a shallow baseline shift in the DSC scan of the polymer at 111 °C, which they attributed to T_g_. Luebbert et al. [130] determined the T_g_ of different low-molecular-weight (MW) HPC grades via extrapolating the glass transition of spray-dried HPC/copovidone blends. The T_g_ varied from 81.6 to 84.2 °C with increasing MW. The complex morphological structure of HPC might account for the difficulties in determining the T_g_ of HPC. Anyhow, the low T_g_ of HPC results in low melt viscosity and fast melt-flow properties (depending on the MW of the polymer); thus, HPC enables formulators to prepare filaments and print them at a relatively low temperature without the help of a plasticizer. Low-molecular-weight grades are processable at temperatures as low as 120 °C, while high-molecular-weight grades are processable at 200 °C without the use of a plasticizer. In addition, extruding at different temperatures and molecular-weight grades also affects the toughness and flexibility of HPC [131]. All these properties make HPC a suitable polymer for melt extrusion-based AM techniques.

Melocchi et al. [64] prepared capsule-shaped devices for the pulsatile release of acetaminophen using HPC with an approximate MW of 95 kDa. The preparation of filaments was conducted at temperatures between 50 and 165 °C based on the concentration of HPC in the formulation. The 3D-printed hollow devices made from HPC filaments showed a pulsatile release behavior with a lag time of approximately 70 min, after which the drug release was completed within 10 min. Ghanizadeh Tabriz et al. [63] successfully printed an isoniazid/HPC (25/75, *w*/*w*%) filament into tablets at 130 °C. Compared to Melocchi et al. [64], the grade of HPC used had a lower molecular weight (only approximately 80 kDa), and the authors also concluded that the filaments prepared by plain HPC were too soft to be fed into a 3D printer. While Henry et al. [40] were able to print the same grade of HPC at 160 °C with no speed restrictions, Zhang et al. [52] observed printing difficulties when evaluating drug-loaded low- and high-MW HPC filaments for their mechanical and rheological properties. The authors observed that the HPC EF and HF filaments were too soft, meaning that they were not strong enough to push the molten material out of the nozzle when fed into the 3D printer. Vo et al. [46] selected copovidone as a co-matrix-forming polymer using a cinnarizine/HPC mixture that was too soft to be printed, causing the filament to be squeezed under the push of the feeding gear of the FDM printer. The combination of HPC and copovidone significantly improved the texture properties of the extruded filament and made it printable. Ideally, filaments for FDM printers should be adequately stiff and brittle, as these properties can help achieve optimal feeding and successful printing. Zhang et al. [53] showed, in a different work, that blending HPC with other cellulose ethers such as HPMC or ethyl cellulose (EC) helped to enhance the filaments’ mechanical properties, making them suitable for the printing process. A similar approach was adopted by Ayyoubi et al. [39], who prepared modified PVA commercial filaments loaded with HPC by passive diffusion (PD) and copovidone or ethyl cellulose by hot-melt extrusion (HME).

#### 3.1.4. Hydroxyethyl Cellulose (HEC)

Hydroxyethyl cellulose is a cellulose ether prepared from the reaction of alkali cellulose and ethylene oxide. Each added hydroxyethyl group introduces a reactive secondary hydroxyl group that can be further etherified during the manufacturing of HEC. Like HPC, HEC can be further substituted, resulting in an additional chain extension. HEC has found widespread use in pharmaceuticals as a hydrophilic matrix-former, binder, thickener, and film-former, and its ease of solubility (both in hot and cold water as well as many organic solvents) makes it an appropriate candidate in many biomedical applications, e.g., different grades of HEC with varying MWs and viscosities have been successfully used to adjust the viscosity of 3D-printing inks. Gospodinova et al. used HEC-based bioinks in an extrusion-based 3D bioprinting process. HEC-containing hydrogels were prepared at a concentration of 5% (*w*/*v*), and sodium alginate (SA) was added in concentrations of 1%, 2.5%, and 5% (*w*/*v*), respectively. Extrudability and shape fidelity served as parameters to assess the printability of the hydrogels. Extrudability in this context refers to the lowest pressure at which the hydrogel could be extruded with a reasonable flow rate for the printing process [59].

Elbl et al. [60] used different grades of HEC as thickening agents for a modified FDM technique, in which the FDM extruder was replaced by a linear syringe pump. Low viscosity and surface tension of the printing dispersion can cause insufficient uniformity of the drug content and unsatisfactory mechanical properties of films due to uneven distribution of the ingredients in the ink paste. The different MW grades of HEC were used with a concentration of 1% (*w*/*w*), leading to printable films with a smooth surface [60]. Luo et al. used 3D-printing technology to fabricate bilayer films comprised of chitosan (CS) and HEC. The best-performing ink was identified to have a CS/HEC ratio of 3:3 and a suitable apparent viscosity, resulting in a consistent printing process with no breakage or clogging observed [61].

Compared to HPC and EC, and even HPMC, the poor thermoplastic behavior of HEC might explain the lack of available studies for HEC in thermal manufacturing processes, such as FDM-based 3D printing. Hartzke et al. [57] evaluated the processability of different grades of HECs in FDM printing and figured out that extrusion of HEC alone was not possible. To overcome this process limitation, the authors mixed HEC (75% *w*/*w*) with thermoplastic HPC (20% *w*/*w*) to improve the overall thermoplastic behavior of the mixture. Together with diclofenac (5% *w*/*w*) as a model API, the ternary mixture enabled the successful FDM printing of tablets. This demonstrates the importance of blending polymers with different properties to facilitate processability based on the requirements of the AM process. The authors concluded that low-viscosity HEC of grade 250 L with a low MW was the most suitable, resulting in sufficient feedability and printability of the filament and the high breaking force of the fabricated tablets [57]. Fina et al. [58] included high-MW-grade HEC 250H as a matrix-forming suspending agent in a formulation comprised of HPC, polyethylenoxide (PEO), and mannitol. During dissolution of the tablet, the dissolution medium dissolves both mannitol and hydroxypropyl cellulose, resulting in void spaces inside. This allows PEO to swell and, thus, create a microenvironment where the drug is suspended and diffuses out slowly. HEC was included at a level of 5% *w*/*w* in the core composition to prevent drug sedimentation [58].

#### 3.1.5. Ethyl Cellulose (EC)

Ethyl cellulose (EC) is a partly O-ethylated cellulose ether derivative that is manufactured by the reaction of alkali cellulose with ethyl chloride at approximately 60 °C for several hours [132]. The substitution level or the ethoxyl content directly impacts the properties of the resulting EC. A typical structure of EC has a DS value of 2.5 per anhydroglucose unit, corresponding to a 48.5 wt.% ethoxyl content. Currently, the major pharmacopeias (USP/NF and EP) define the ethoxyl content of EC to be between 44.0 and 51.0% (*w*/*w*), and various ethyl cellulose types are distinguished by their molecular weight or viscosity, respectively (Table 4). Like other cellulose ethers, the physical properties of EC depend on the MW, its degree of etherification, and the distribution pattern of the substituted groups.

EC is considered to be a biodegradable material, and in contrast to most other cellulose ethers, it is not soluble in water but can be dissolved in most organic solvents, and thus, EC-containing printing inks are solvent-based. Adams et al. dissolved different amounts of EC in an alpha-terpineol solvent to vary the viscosity of the resulting printing inks. The authors customized a direct ink writing printer for printing the EC solutions and demonstrated a steady flow for a range of EC inks with different viscosities, suitable for 3D printing. This process was utilized to successfully fabricate biopolymer parts [54].

Kavimughil et al. formulated an 11% (*w*/*w*) EC-based oleogel using medium-chain triglyceride and stated acceptable printability via an oil-binding capacity test. The authors assessed printability at different temperatures, and 45 °C was reported to provide the best printability and process performance. The study highlighted the potential of oleogel systems for AM applications, with the improved bioaccessibility and bioavailability of hydrophobic/lipophilic active ingredients [133].

Yu et al. [47,48,49] used ethyl cellulose among other polymers in DoP binder jetting applications. In one of the cases [49], EC was used to print multi-layered doughnut-shaped drug-delivery devices, where the API was sandwiched between ethyl cellulose-rich, drug-free top and bottom barriers, enabling a linear drug-release profile. EC served as the retarding polymer controlling the drug dissolution rate; however, it also helped to provide strong adherence forces with the drug-loaded regions in the printed shape and eliminate the burst effect in dissolution testing.

EC exhibits a relatively high T_g_ at 132 °C, has a melting point T_m_ of around 180 °C (depending on the polymer MW), and its storage modulus remains in a glassy state up to 64 °C, indicating rigid filaments at room temperature. Therefore, EC is a suitable polymer for FDM filament printing that exhibits sufficient thermal plasticity and, thus, extrudability. In a study performed by Zhang et al. [53], modified-release tablets were prepared by FDM coupled with HME to fabricate filaments. The results indicated that the EC filaments were too brittle, and thus, they were broken by the FDM printer’s feeding gears during feeding. To improve the brittleness of the EC filaments, EC was mixed with other polymers, such as HPC, HPMC, copovidone (PVPVA), poly(vinyl alcohol), polyethylene glycol, or xanthan gum. Mixing EC with those polymers helped to adjust the EC filaments’ stiffness and brittleness and led to well-extrudable mixtures and good printable filaments [53]. Borujeni et al. mixed EC and HPC and evaluated the effect of different blends on the mechanical properties of extruded filaments and their printability. The best properties for FDM printing were achieved by a filament formulation containing CBZ, EC, and HPC (3%, 64.7%, and 32.3% *w*/*w*, respectively). This filament resulted in 3D-printed tablets with appropriate mechanical properties and good content uniformity in the API [55]. Shi et al. fabricated ibuprofen and EC-based matrix systems, combining various polymers and extruding them into filaments by twin-screw extrusion. The filaments were successfully printed using an FDM printer, and the mechanical characterization indicated that the filaments’ stiffness and brittleness were significantly improved by adding other polymers to the EC/ibuprofen matrix. Furthermore, in vitro dissolution studies showed that it was possible to control the drug release over 24 h by varying the additional polymer and its hydrophilicity [38]. Yang et al. [56] evaluated EC-based formulations to sustain the release of ibuprofen. Ratios of 50–80% (*w*/*w*) EC and 16–24% ibuprofen were blended with additional release modifiers such as HPMC, sodium alginate, xanthan gum, or polyvinyl alcohol before hot-melt extrusion at a 100–120 °C processing temperature. The FDM printability was greatly affected by the melt rheology and mechanical property of the filaments fed into the FDM printer; however, the targeted drug release within 24 h was achieved by an optimized formulation and printing process parameters affecting the infill pattern and density, as well as the shell thickness of the printed shapes. In conclusion, EC is a suitable material for HME/FDM 3D printing. Generally, lower-viscosity EC grades tend to show better thermoplastic properties than higher-MW grades, probably due to better alignment and less steric hindrance in low-MW grades [27,117].

### 3.2. Polyvinyl Polymers

**Figure 4:**
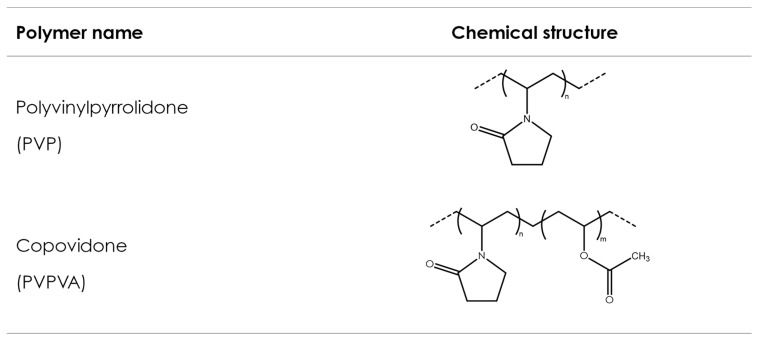
Polyvinylpyrrolidone and copovidone.

Polyvinyl polymers are synthetic amorphous linear polymers synthesized from formaldehyde and acetylene using the Reppe process to obtain N-vinylpyrrolidone (NVP) over different synthesis steps [134]. NVP is consequently polymerized either alone (to obtain homopolymers) or with other monomers (to obtain copolymers) using a free-radical polymerization step, induced by the addition of an initiator, which controls the final molecular weight of the synthesized polymer. Polyvinylpyrrolidone (PVP) is obtained from the polymerization of NVP solely, while vinyl acetate-vinylpyrrolidone copolymer (PVPVA) is obtained from the polymerization of NVP with vinyl acetate. Figure 4 shows exemplary chemical formulas for both polymers. Both polymers show favorable physicochemical characteristics, such as solubility in a wide variety of solvents, including water; ability to interact both with lipophilic and hydrophilic substances [135]; good adhesion properties; thermoplasticity; and low toxicity [136]. Consequently, they have been broadly used in pharmaceuticals—for example, as tablet binders [137], thickeners, and in the solubilization of drugs by forming amorphous solid dispersions [138]. In pharmaceutical AM, PVP and PVPVA have been used as suitable excipients in FDM, SLS, and binder jetting technologies.

#### 3.2.1. Polyvinylpyrrolidone (PVP)

Polyvinylpyrrolidone (PVP) is also known by its compendial name povidone. It is available in different molecular-weight grades, reflecting the number of repeating vinylpyrrolidone units. Historically, difficulties in the determination of the molecular weight of this polymer led to the introduction of the *K*-value concept, as a reference to the molecular weight. The *K*-value, also known as the Fikentscher viscosity coefficient, is calculated from the kinematic viscosity of an aqueous polymer solution, measured using a capillary viscosimeter [139], and its concentration:

(1) $$log\frac{\eta_{rel}}{c}=\frac{75K_{0}^{2}}{1+1.5K_{0}c}+K_{0}$$

In this equation, the relative viscosity of the aqueous solution compared to that of the solvent is represented by *η_rel_*, the concentration of the solution in g/100 mL is given by *c*, and *K*_0_ is the *K*-value multiplied by 10^−3^. The Fikentscher equation can also be rearranged, and thus, the *K*-value can be directly calculated, as shown in Equation (2):

(2) $$K=\frac{\sqrt{300c\log\eta_{rel}+\left(c+1.5c\;log\eta_{rel}\right)²}-c+1.5clog\eta_{rel}}{0.15c+0.003c^{2}}$$

Different pharmacopoeias, such as the USP-NF, EP or JP, accept that the K-value calculated by Fikentscher’s equation indirectly represents the molecular weight (MW) of the polymer and the viscosity of its solutions. Still, other methods such as gel permeation chromatography, light scattering, and vapor pressure osmometry have been used for determining the MW, and thus, the resulting representation of MW would be different depending on the used method of determination (weight average, M_W_; intrinsic viscosity; or number average, M_n_) [134,140]. Table 5 shows different grades of PVP along with their typical *K*-value ranges and molecular weights, determined with different methods.

The solution viscosity, glass transition temperature, and adhesive properties of the polymer typically increase with increasing molecular weight. On the contrary, the dissolution rate of the polymer increases when the molecular weight decreases, due to the viscosity reduction of their solutions. The melting point temperature (T_m_), glass transition temperature (T_g_), melt index, and thermal degradation temperature (T_deg_) are fundamental characteristics related to the thermoplastic behavior of polymers. Table 5 illustrates the relationship between the T_g_ and molecular weight or *K*-value, respectively. Similar to the T_g_, melting temperatures and melt viscosity increase with increasing molecular weight [22]. These polymer properties are also relevant for some 3D-printing technologies, e.g., FDM printing. For melt-based process technologies, the polymers should possess thermoplastic properties to allow printing; however, they need to be processed in temperature ranges in which the polymers remain thermally stable. Therefore, polymers with a wide thermal processing window (typically the temperature range between the polymer’s T_g_ or extrudability temperature and its degradation temperature) should be selected for optimized filament feeding. Still, the use of PVP in FDM remains challenging due to its limited extrudability in the absence of an appropriate plasticizer or other ingredients with a plasticizing effect on the polymer. The resulting filaments are often brittle, causing difficulties in the feedability of the filament into the printer.

Numerous studies describe the use of PVP in 3DP. Kollamaran et al. [41] combined a PVP (low-molecular-weight grade) with copovidone in formulations containing ramipril, with the aim of achieving an immediate-release (IR) profile of the printed drug product. The addition of the low-MW PVP lowered the necessary printing temperature during the FDM process, allowing for the printing of a thermolabile and low-melting drug like ramipril without degradation. Moreover, the formulations combining low-MW PVP with copovidone showed a slightly faster drug release than those containing just copovidone.

Okwuoasa et al. [101] fabricated immediate-release tablets containing two drugs (dipyramidole and theophylline) in a dual FDM printing process. The processed filaments contained each drug alone, PVP, and the thermally stable filler talc. The printing process was conducted using a temperature of 110 °C, which is regarded as relatively low. In another study, the same authors prepared tablets with delayed-release properties containing PVP and the enteric polymer methacrylic acid-ethyl acrylate copolymer (MAAEA), also using a dual printing FDM process using two different filaments. In this case, the enteric shell of the printed tablets was made from MAAEA while the IR core contained theophylline dispersed in PVP [102]. Kempin et al. [80] provided a further example of using a dual extrusion FDM printing process. While the outer shell was made from filaments containing enteric polymers, PVP was used in the tablet core.

As mentioned before, as the molecular weight of the polymer increases, the solution viscosity increases (Table 5). PVP is water soluble, its maximal solubility being limited only by the viscosity of its own solution. Compared to most cellulose ethers, solutions of low-MW-grade PVP have an exceptionally low viscosity. PVP is also nonionic and its viscosity in aqueous solutions is not affected by the pH or by salts. These properties allowed the preparation of an aqueous PVP-based ink for solvent inkjet printing (DoD) [23].

Due to its properties, PVP is also an important excipient in binder jetting technologies. One of the first publications using PVP in this application combined it with small amounts of maltitol and maltodextrin to print fast-dissolving tablets of hydrophilic captopril [94]. The first ever approved 3D-printed drug, Spritam^®^ (Aprecia Pharmaceuticals, Blue Ash, OH, USA), was also prepared using binder jetting and PVP [4]. While the tablet matrix contains 60–90% (*w*/*w*) of the drug levetiracetam along with microcrystalline cellulose, mannitol and colloidal silicon dioxide, the matrix materials are bound together by an aqueous solution of PVP, glycerin, and a surfactant. Tablets prepared in this manner contain twice as much API compared to conventionally manufactured tablets. The fabricated tablets are highly porous, resulting in a short disintegrating time, leading to a noticeable improvement in patient compliance. Studies comparing different binders in similar process conditions were conducted by Lee et al. [94] and Tian et al. [100]. The results of both studies indicated that 3D-printed tablets containing PVP had an appropriate tablet hardness; however, their disintegration time was longer than expected. Another study using a DoP printing process conducted by Sen et al. [96] focused on the mechanical strength of printed tablets containing amitriptyline HCL as a model drug. Combinations of eight fillers and ten binders were studied. The formulation comprised of lactose monohydrate and PVP K30 increased the mechanical strength of the printed tablets, most likely due to the formation of solid bridges. The authors also observed that not all tested binders provided the necessary strength to the tablets, with PVP being one of the few binders that performed well. Similar findings were reported by Tian et al. [95], who prepared orally disintegrating tablets containing warfarin as an API. They observed that the combination of D-sucrose and the PVP K30 as the filler and binder resulted in printed tablets with sufficient mechanical properties and an appropriate disintegration time. Kozakiewicz-Latała et al. presented a formulation approach that allowed for the manufacture of fast-dissolving tablets with a small dose (3 mg) of the hydrophilic model API quinapril hydrochloride. Mixing the API with microcrystalline cellulose as a bulking agent and PVP K25 as a binding agent led to porous fast-dissolving tablets with satisfactory mechanical properties [97].

Povidone has been used in PAM printing processes as well, e.g., Dores et al. [106] compared tablet formulations containing 40% (*w*/*w*) theophylline as a drug; PVP or PVA as a polymer; and lactose, D-Mannitol, and sorbitol as highly soluble fillers/plasticizers. Tablets containing PVP and lactose tend to show a more cohesive structure compared to tablets made with PVA. In another study, Khaled et al. fabricated polypills consisting of different compartments to adjust the release profiles of three APIs [71]. PVP was used as a binder for the immediate-release compartment. Abdella et al. [75] demonstrated that PVP can be used to prevent the separation/sedimentation of the drug from the suspension used during the printing process. The addition of PVP increased the viscosity of the printed mucoadhesive buccal film and, thus, helped suspend the API in the film-forming solution.

#### 3.2.2. Copovidone (PVPVA)

Copovidone (PVPVA) is a random, linear copolymer produced by the free radical polymerization of vinylpyrrolidone and vinyl acetate (VP–VA copolymer; Figure 4) with a 6:4 ratio of VP to VA. It is a freely flowing, spray-dried powder with a spherical, hollow-particle morphology and was designed to overcome some of the limitations associated with polyvinylpyrrolidone. PVP, for example, is a relatively stiff, brittle, and hygroscopic material, although it is frequently used as a tablet binder. The brittleness and stiffness are reflected in the relatively high T_g_ of approximately 164 °C for a PVP (PVP K30), compared to a lower T_g_ of approximately 108–111 °C for PVPVA, with a molecular weight close to the MW PVP K30. The decrease in the T_g_ of copovidone due to the addition of a comonomer to vinylpyrrolidone improves the copolymer plasticity and flexibility, and thus, PVPVA has a significantly lower T_g_ and is a lot more flexible and plastic compared to PVP (Table 6).

While most material properties of polymers, such as the MW, DS, nature of the functional groups, etc., are imparted by the synthesis step of the manufacturing process, there are also properties imparted after the synthesis (due to drying or milling), such as particle size, density, and particle shape. These properties can impact material characteristics such as powder flow and powder handling and are important for the processability of the polymers. As an example, the original, regular grades of copovidone (Plasdone™ S630 and Kollidon™ VA64, respectively) were improved by introducing a finer particle-size grade (Kollidon™ VA64 fine) for better tablet binding, e.g., in dry granulation by roller compaction [143], or by adjustments in powder flowability and particle size distribution, resulting in better processability in hot-melt extrusion processes (Plasdone™ S630 ultra) [142].

Furthermore, copolymerizing vinylpyrrolidone with vinyl acetate also reduces the hygroscopicity of PVPVA compared to PVP [134], resulting in up to three times less water absorption compared to PVP [146].

The combination of good thermoplastic properties and low T_g_ make copovidone a suitable polymer for melt-based processes (e.g., hot-melt extrusion), and, therefore, copovidone has been chosen for FDM processes as well. However, the inherent high brittleness of PVPVA can pose challenges to some printing processes. Henry et al. [40] tried to print copovidone filaments in FDM printing; however, they failed at successful printing because of this. From hot-melt extrusion (HME) applications, it is known that the processability of brittle polymers such as PVPVA can be significantly improved by combining them with plasticizers or other polymers, and consequently, this was adopted for FDM printing as well. Kollamaram et al. added a plasticizer, PEG 1500, to a PVPVA-based formulation to lower its T_g_, and thus, they improved the processing performance of the formulation [41]; Solanki et al. [42] mixed PVPVA and HPMC to improve the mechanical properties of the filament and enable a successful printing process. In addition to plasticizers or other polymers, APIs can help to enable the printing of copovidone-based filaments as well, due to their plasticizing effect on the formulation. Ayyoubi et al. demonstrated a successful fabrication of spherical 6 mm mini tablets combining copovidone with nifedipine. A drug loading of 50% nifedipine helped to produce filaments by hot-melt extrusion that were able to be fed into the FDM printer [39]. Boniatti et al. [9] explored the use of direct powder extrusion to reduce the dependence on the strict mechanical properties of HME filaments for FDM printing. Tablets contained PVPVA and 35% or 50% (*w*/*w*) API, respectively, and were evaluated with a special focus on the drug dissolution profiles, physical stability, and taste-masking effectiveness.

Copovidone has also been utilized in binder jetting technologies. Chang et al. [98] reported the remarkably better performance of copovidone compared to PVP with respect to the binding strength and disintegration behavior. Gottschalk et al. used a drop-on-powder printer to fabricate tablets containing poorly water-soluble ketoconazole embedded in a PVPVA-based amorphous solid dispersion. While the amorphous solid dispersion was prepared via hot-melt extrusion with drug loadings of 20% and 40%, the actual printing took place after milling the extrudates to powdered material again. This approach can serve as an alternative to FDM printing and help to overcome the limited mechanical properties of the copovidone-based feedstock [31].

Selective laser sintering (SLS) is another technology where copovidone has been used with success: Fina et al. printed tablets with fast disintegration behavior using copovidone and low-dose paracetamol as a model drug (5% (*w*/*w*)). The authors investigated the influence of several printing parameters on the drug-release characteristics of the printed formulations. A faster laser scanning speed resulted in tablets that were completely dispersed in less than 4 s in a small volume of water, like conventional manufactured orally disintegrating tablets (ODTs). The authors concluded that the less-energetic sintering process at a faster laser scanning speed caused the powder particles in the fabricated tablet to better separate from each other upon contact with liquids, resulting in the faster disintegration time of the printed tablets. Furthermore, the higher porosity of tablets fabricated at faster laser scanning speeds shortened the disintegration time, as the dissolution medium was able to penetrate the porous tablet structure through capillary action, also commonly referred to as “wicking” [33]. Barakh Ali et al. [36] investigated the effects of formulation and process variables on the quality of diclofenac sodium-loaded printlets using SLS. The formulations consisted of 55–59% (*w*/*w*) PVPVA, 30% (*w*/*w*) API, 8–12% (*w*/*w*), and 3% (*w*/*w*) Candurin^®^ NXT Ruby Red. The latter material was included to enhance the energy absorption from the laser and thus aid the printability of the formulation. Surface temperature, laser scanning speed, and lactose concentration were studied for their effect on the printlet quality, with all of them showing significant impact. The authors concluded that it was possible to fabricate printlets with good mechanical integrity and fast disintegration and dissolution rates using SLS. Allahham et al. [37] used PVPVA and Mannitol to print drug–cyclodextrin complexes. At a 100 °C printing temperature, copovidone reaches a rubbery state, and polymer particles connect to each other, forming bridges and sintered areas following the passage of the laser. While PVPVA already enters a rubbery state due to its T_g_ of around 109 °C at the printing temperature, Mannitol has a melting point of approximately 168 °C, and thus, the Mannitol particles either dissolve in the rubbery PVPVA or are unmodified, trapped within the PVPVA matrix. This combination allows for the fabrication of printlets with a high porosity, resulting in fast disintegrating times. Awad et al. [11] used 92% *w*/*w* PVPVA due to its good printability and fast disintegration properties. The authors manufactured personalized printlets with Braille or Moon patterns on their surface, targeting blind or visually impaired individuals. Mohamed et al. utilized SLS printing to fabricate tablets containing clindamycin palmitate HCl along with PVPVA, microcrystalline cellulose, and lactose monohydrate. The laser scanning speed was concluded to be the most important process factor, directly impacting the porosity of the tablet, thus impacting the dissolution rate and tablet disintegration as well as the tablet breaking force and crystallinity of the printed tablet [34]. Davis et al. [35] used a single-step SLS process to print copovidone-based amorphous solid dispersions with Ritonavir as a model drug. Process parameters affecting the melting of the composition such as surface temperature and hatch spacing were identified to have a significant impact on the ability to fabricate a fully amorphous product.

### 3.3. Bioresorbable Polymers—Aliphatic Polyesters

Aliphatic polyesters are a class of bioresorbable polymers that have been widely studied and employed in recent years [147]. Biodegradability is achieved due to an aliphatic ester bond on the polymer backbone, which is hydrolyzed in aqueous environments. Moreover, their degradation by-products are eliminated from the body via natural metabolic pathways, making these materials attractive for biomedical applications.

**Figure 5:**
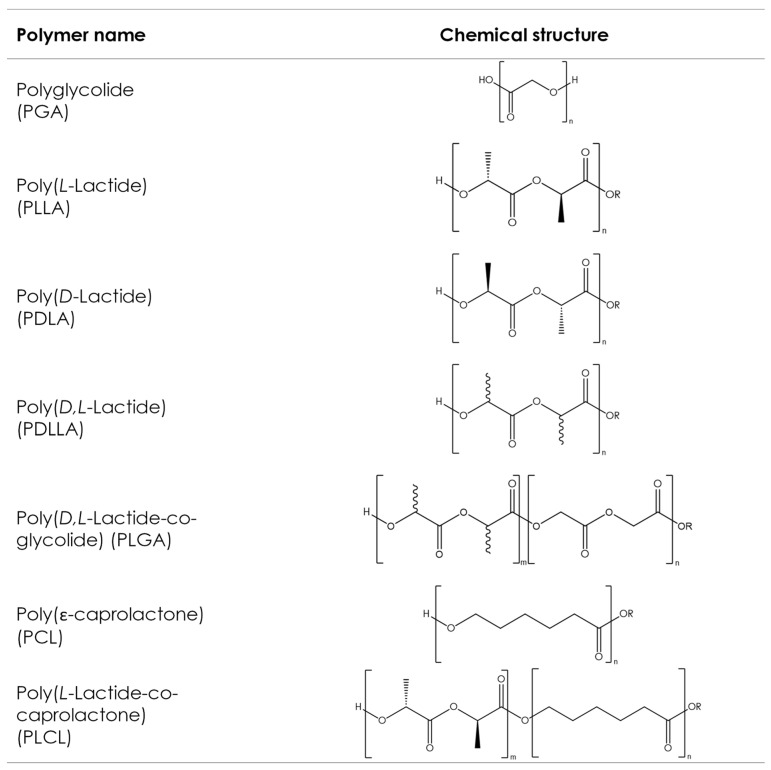
Chemical structures of the commonly used aliphatic polyesters. Bonds with wavy lines indicate that the stereochemistry of the bond is unknown.

Although various types of aliphatic polyesters exist, such as naturally derived polyhydroxyalkanoates (PHAs) or poly(alkenedicarboxylate)s, this article focuses on the most commonly used synthetic aliphatic polyesters, often described as poly-α-hydroxy acids or poly-α-hydroxyesters, whose chemical structures are shown in Figure 5. These polymers have been used in a medical context for more than 50 years and are present in a large variety of medical products commercially available on the market with various applications, like implants for prolonged drug delivery, long-acting injectables (LAI) for extended drug release, orthopedic fixation devices, wound dressings, and scaffolds for tissue engineering [148,149]. However, such products put little focus on customization and, in general, take a one-size-fits-all approach. AM processes, such as 3D printing, make customization possible by allowing the production of dosage forms or implants with complex geometries, which fit the patient’s individual anatomy in a quick, cost-effective manner without sacrificing precision [150].

The ring-opening polymerization (ROP) of cyclic di-ester monomers is the most commonly used method of synthesizing poly-α-hydroxyesters. While the polycondensation of respective difunctional acids can also be used [151,152], pharmaceutical applications typically require relatively high molecular weights, and this can only be easily achieved via ROP [153,154]. The physical, mechanical, and processing properties of these synthetic polyesters are mainly influenced by the type of monomers used and the final molecular weight [155], both of which are easily controlled during synthesis.

This ability to easily tailor or tune material properties, and their inherent thermoplastic nature, make aliphatic polyesters excellent FDM printing materials. However, aliphatic polyesters are not thermally stable, and FDM-style printing methods at excessively high processing temperatures can lead to the onset of degradation and a drastic change in material properties. The presence of moisture during processing can also onset degradation and, consequently, a drying step is typically conducted prior to thermal processing. In general, thermal processing should be conducted at the mildest temperatures possible, with moisture excluded from the process as thoroughly as possible. As well as FDM, aliphatic polyesters have also been utilized in DoP-, DoD-, and PAM-style 3D printing due to their solubility in a variety of organic solvents, such as acetone, chloroform, dichloromethane (DCM), and N-methyl-2-pyrrolidone (NMP), as well as binary solvent mixtures like acetone/ethanol or ternary mixtures like DCM/acetone/ethanol [156]. Regardless of the printing methodology utilized, it is crucial to understand the physicochemical properties of the polymer before and after printing to achieve the desired performance in the relevant pharmaceutical dosage form. Table 7 shows the most relevant material properties of the aliphatic polyesters used in pharmaceutical 3D printing.

#### 3.3.1. Polyglycolide or Poly(glycolic acid) (PGA)

PGA is considered to be a thermoplastic, rigid polymer showing comparably high crystallinity (45–55%) and a degradation time frame of between 6 and 12 months [157,160]. Because of its limited solubility in most organic solvents, its use is limited in pharmaceutical applications. However, PGA is often copolymerized with other bioresorbable monomers, such as lactide or caprolactone, or it is blended with other polymers to enhance their mechanical properties or impact degradation rates. Zhang et al. tailored the stiffness–toughness mechanical performance of PGA by blending it with poly (butyleneadipate-co-terephthalate) (PBAT) in their FDM 3D-printing study [159]. The authors used hot-melt twin-screw extrusion to produce composite filaments containing PGA/BAT in various combinations (100/0, 95/5, 85/15 and 75/15) with Joncryl ADR 4375 (1.5% *w*/*w*) at 210–230 °C. The extruded filaments served as the feedstock for FDM printing and were successfully printed at a 230 °C printing temperature. The interaction between PGA and BAT was improved with the addition of ADR. This resulted in a homogeneous polymer network, containing less hydroxyl and carboxyl end-groups in both polymers. ADR was also crosslinked with PGA/PBAT, leading to an increase in the melt strength. Furthermore, the PGA/PBAT formulations (ratios of 95/5 and 85/15, respectively) were thermally stable, unlike neat PGA and PGA/PBAT (75/25), which decomposed.

#### 3.3.2. Polylactide or Poly(lactic acid) (PLA)

PLA is the most popular linear aliphatic polyester and is typically synthesized via the ROP of the cyclic dimers of lactic acid, known as lactide. Lactide is a chiral molecule and exists in three enantiomeric states: L(−), D(+), and meso-lactide [162]. The polymer can be synthesized with only one of the enantiomers (poly(L-lactide) (PLLA) and poly(D-lactide) (PDLA), which are isotactic and semi-crystalline (~37%)) or a racemic mixture of monomers containing an equal number of both L and D enantiomers (poly(D,L-lactide) (PDLLA), which is atactic and amorphous) [163]. The amorphous PDLLA exhibits a markedly lower tensile strength, modulus, and degradation rate than its semicrystalline counterparts [164,165].

In the field of 3D printing, PLA has seen widespread use as a printing material across a multitude of applications and industries. It is often blended with other polymers or additives to obtain 3D-printed composites with more adequate material properties and performance characteristics. For example, Fu et al. [77] FDM-printed personalized vaginal rings containing progesterone (PRG) and mixtures of PLA and PCL to enhance the ductility and flexibility of the relatively brittle PLA, which can present issues during surgical handling and implantation. They investigated formulations containing mixtures of PLLA (10 kDa Mw) and PCL (80 kDa Mw) in various ratios, along with Tween 80 and PEG 4000 as dispersants. Filaments were first prepared using melt extrusion, starting from the polymer blend and a solid dispersion of amorphized PRG in PEG and Tween 80. Filaments with and without drugs were then 3D-printed using a nozzle temperature of 195 °C, a hot-bed temperature of 60 °C, and an extruding speed of 18 mm/s. Blends with up to 30% PCL yielded good-quality bendable filaments, but the quality strongly deteriorated beyond that threshold. The resulting rings successfully released PRG over 8 days, with the rate of release depending on the shape of the rings (O, M, and Y). Personalized vaginal rings can help in avoiding pelvic inflammatory disease and uterine preformation, which can occur from the one-size-fits-all approach to existing market products.

Blends containing PLA and PVA were evaluated by Liang et al. in their study, in which wearable devices in the form of personalized mouthguards were prepared by FDM [78]. The mouth of the test persons was scanned using an intraoral scanner and subsequently used in the 3D printing of highly personalized devices. These devices contained clobetasol propionate (CBS), with the aim of releasing the drug over a few weeks to alleviate oral inflammation. CBS-containing devices (10%) were tested only in vitro, while CBS was replaced with 2.5% vanillic acid (VA) for conducting an in vivo study. Filaments containing various ratios of polymers were produced via melt extrusion, and they exhibited appropriate characteristics for consequent 3D printing. CBS and VA were amorphized in the resulting filaments. The in vitro drug release was assessed in artificial saliva. A drug-release rate of 39% and 19% was achieved for blends containing 33% and 44% PLA, respectively. The entire process took two hours from scanning until the production of the device, which had a good fit anatomically in the mouths of all test persons [78].

Shuai et al. utilized SLS 3D-printing technology to create composite scaffolds for bone repair with blends of PLLA, PGA, and hydroxyapatite (HAP) to boost bioactivity and osteoconductivity [166]. The weight loss of scaffolds made from the polymer blend after immersion in PBS for 28 days was 25% versus only 3.3% for the less-hydrophilic PLLA alone. Consequently, the HAP protruded from the gradually hydrolyzing matrix, promoting the deposition of bone-like apatite in the body during in vivo experiments in New Zealand white rabbits. This accelerated the development and acceleration of osteoblasts, thus facilitating the growth of new bone tissue and blood vessels. The authors highlighted the potential use of 3D printing scaffolds containing PLLA/PLGA/HAP in bone-tissue engineering since they are biodegradable, bioactive, and induce osteogenesis.

#### 3.3.3. Polylactide-co-Glycolide or Poly(lactic-co-glycolic acid) (PLGA)

PLGA is a block copolymer of PLA and PGA and is the most utilized aliphatic polyester in a pharmaceutical context today. PLGA grades used in pharmaceutical applications are predominantly synthesized from racemic D,L-lactide, and most of the used compositions contain 50–85% thereof. As a result, they are typically amorphous polymers and their mechanical properties and biodegradability depend on the ratio of both components. Compared to PGA, PLA is more hydrophobic and resistant to hydrolysis due to the additional pendant methyl side groups [165]. Therefore, the rate of degradation by hydrolysis of PLGA copolymers decreases with increasing lactide content and can range from several weeks to several months [167].

Guo et al. [88] printed PLGA scaffolds using a DoP-style bioplotter with two needle diameters (0.2 and 0.4 mm) at various temperatures (95–170 °C). The authors used PLGA polymers with molecular weights ranging from 10 to 60 kDa; different LA/GA ratios (50:50, 60:40, and 85:15); and ester or acid end-capped polymer chains. Melt rheology measurements were conducted within safe temperature ranges for each polymer composition at a fixed frequency of 10 rad/s. Complex viscosity measurements as a function of temperature revealed that increasing temperature led to a decrease in complex viscosity, with an optimal process window of 0.1–10 Pa allowing for sufficient processability and the best printability. While the scaffolds printed from 50:50 and 60:40 LA/GA compositions exhibited a significantly lower MW compared to the pre-printed polymers, the highest MW composition of 85:15 LA/GA did not show such a behavior. The compressive strength and morphology in scaffolds with a low LA content deteriorated quickly in the first two weeks of in vitro degradation (37 °C in PBS). An ester end cap yielded a more-hydrophobic and slower-degrading polymer than acid end caps [149]. Scaffolds with higher lactide content and an ester end cap were deemed preferable for the retention of the scaffold structure and integrity. The authors concluded that such compositions are suitable for scaffold materials used in cartilage regeneration, due to their relatively slow degradation rate and compressive mechanical strength similar to native human cartilage [168].

Moreover, 3D printing allows for the fabrication of complex devices containing multiple APIs, which opens the door to combination therapies for specific applications or indications. For example, Qiao et al. printed PLGA scaffolds for the delivery of both doxorubicin and cisplatin concomitantly using an E-jet 3D-printing process [87]. An adapted printing system with a high-voltage nozzle was used to print porous scaffolds from a solution of PLGA comprised of 75:25 LA/GA and both drugs in DMF. Residual solvents were subsequently removed from the scaffolds by lyophilization for 24 h under vacuum and the scaffolds were analyzed in vitro and in vivo (in mice) afterwards. The results indicated a sustained drug release for 30 days while increasing cancer cell apoptosis and inhibiting tumor growth. Moreover, the combination therapy allowed for a decreased overall drug dosage compared to each drug administered alone without the polymer scaffold. Drug release can be further tweaked in such implants by changing the in-filling percentage during the printing process. Bassand et al. [169] produced implants containing PLGA (LA:GA 50:50, MW 39 kDa, ester end group) and ibuprofen using a droplet deposition modeling method at temperatures of 100–120 °C, with a nozzle diameter of 250 µm and a printing speed of 40 mm/s. The printed implants had the shape of parallel piped meshes and the filling density was set to 10%, 30%, or 100%. Drug release from the implants was measured either in agarose gels or in phosphate buffer (pH 7.4) and was found to be the same at filling densities of 10% and 30% irrespective of the test media, with a complete drug release after 40 days in the gel versus 12 days in the buffer. Similarly, the release was biphasic in the buffer, with the first release phase ending after 5 days, while it was monophasic in the agarose gel. Implants with 100% theoretical filling released more slowly than those with 10% and 30% filling, releasing slowly in the first phase (10 days), then faster in the second phase, with a complete drug release after 40 days—like the first two cases, only with different kinetics. The authors concluded that the drug release depends on the existence of an interconnected aqueous phase. The drug release remains similar until the porosity becomes so low that the release medium cannot build a continuous network. The drug must consequently diffuse through the swollen polymer of the entire implant, slowing down the drug release.

Serris et al. utilized a DoD-style ink-jetting process to fabricate various PLGA-containing films. Using different APIs, the authors prepared single-layered, tri-layered, and core-in-shell PLGA films [86]. The use of this technique enabled making films with specific shapes and structures on demand—something that cannot be done via traditional solvent casting methods. A syringe was filled with an acetone solution containing PLGA polymers and APIs and then printed. The authors studied two PLGA polymer compositions of either high MW (50:50 PLGA; 150 kDa) or low MW (50:50 PLGA;12-16 kDa), and a water-soluble PEGylated tri-block hydrogel composition of PLGA–PEG–PLGA (1.5/1/1.5 kDa). While films containing lidocaine as a model drug showed a constant release rate, Paclitaxel- and rapamycin-incorporated PLGA films displayed delayed release patterns. The same APIs exhibited a more rapid release from the PLGA–PEG–PLGA polymer composition, with a shorter half-time compared to the PLGA films. This can be attributed to the molecular weight and hydrophilicity of the PLGA–PEG–PLGA polymer compared to the PLGA counterparts [170].

#### 3.3.4. Polycaprolactone or Poly(ε-caprolactone) (PCL)

Like PLA, PCL is an aliphatic polyester that has seen widespread use across various industries and applications. PCL degradation can take up to two to three years under physiological conditions, and the degradation process is characterized by a slow decrease in MW without deformation [171]. PCL is soluble in a wide range of organic solvents and has a low T_m_ and T_g_ of 60 and −60 °C, respectively. This makes PCL highly processable and suitable for use in different printing techniques. In addition, these thermal properties make PCL a semi-rigid, ductile material at room temperature [172]; thus, PCL is one of the most preferred polymers for extrusion-based 3D printing [173]. Radhakrishnan et al. [83] created PCL/silver nanoparticle (AgNps) composites that were extruded into filaments further used for the fabrication of customized 3D porous scaffolds by FDM printing. PCL has a relatively low tensile strength and a Young’s modulus of between 20–34 and 206–345 MPa, respectively, as well as a very high elongation at break [160]. The presence of AgNps in the PCL scaffold improved its stiffness (Young’s modulus) and enzymatic stability [83], resulting in scaffolds with good mechanical properties, high flexibility, and great elongation.

Viidik et al. used hot-melt-extruded PCL filaments for the FDM printing of tablets [82]. Investigated compositions contained physical powder mixtures of PCL, Gum Arabic as a plasticizer, and indomethacin or theophylline as an API. The formulations were converted into filaments by a single-screw extruder and consequently printed by an FDM system. The formulation containing PCL and Gum Arabic enabled the incorporation of up to 40% (*w*/*w*) of both APIs into the HME filaments. The extruded filaments were easily printable into tablets with a smooth surface and sufficient mechanical properties at relatively low temperatures of 75 °C. This allowed for the incorporation of temperature-sensitive APIs or other substances. Kim et al. used an FDM-type 3D printer [81] to develop biodegradable stents of heat-sensitive antibiotics amoxicillin and cefotaxime from PCL with a MW of 45 kDa. The use of PCL allowed for a processing and printing temperature of 70 °C, and thus, both APIs maintained their antimicrobial activity post-printing. The produced stents showed a sustained release of the antibiotics, rendering them useful for treating the causative bacteria.

A pressure-assisted micro-syringe (PAM) 3D-printing process was recently used by Rahman-Yildir et al. to create PCL-based inserts for targeted drug delivery in the bladder [84]. Three APIs were incorporated into implants: lidocaine hydrochloride, trospium chloride (TrCl), and hydrochlorothiazide (HCT). The implants were fabricated using biodegradable PCL and non-degradable ethylene vinyl acetate copolymer (EVA). The drug release of the implants was affected by the formation of API clusters inside the matrix and the solubility of the API in the dissolution medium. The combination of PCL–TrCl showed the fastest drug release within 15 days, with the potential to offer customized release profiles by varying the drug doses between 5% and 15% and by altering the printing geometry of the inserts.

Won et al. prepared core/shell rods containing two different APIs to be released from a single implant using a multi-head bioprinting technique [174]. The coaxial printing process enabled the authors to fabricate rod-like implants with a shell consisting of PCL and bevacizumab and a core based on alginate and dexamethasone. The implants were injected in the rat vitreous and enabled different drug-release kinetics at the same target site, due to the different polymers used. While dexamethasone was released within 7 days, PCL enabled a controlled release of bevacizumab for 60 days, respectively.

#### 3.3.5. Poly(L-lactide-co-ε-caprolactone) (PLCL)

The copolymerization of lactide and caprolactone monomers yields PLCL, a copolyester that combines the ductile mechanical properties of PCL with the faster degradation and biocompatibility of PLA. Copolymerization with PCL results in a drastic reduction of the glass transition temperature and crystallinity of neat PLA, yielding a gradual transformation from a glassy, thermoplastic material with a clear yield point to a material exhibiting elastomer–thermoplastic behavior that is more deformable [161]. PLCL polymers are more resistant to thermal degradation compared to PLLA, although the onset temperature of their degradation is lower (all >190 °C). This implies that a PCL content of at least 20% would render the manufacturing of pliable filaments for use in FDM printing easier, without considering the effect of the addition of other components like APIs, which often have a strong impact on the mechanic characteristics of the filaments [89,90]. Bachtiar et al. [90] found that the amount of the crystalline portion (coming from the PLLA portion) has an impact on the mechanical properties of the resulting polymers, which are considerably stiffer. Moreover, 3D-printed scaffolds containing pure PLCL (60:40) and PLA/PCL (70:30) were achieved in an FDM printing process using the following process parameters: 180 °C nozzle temperature, 0.4 mm nozzle size, printing speed of 6 mm/s, and a bed temperature of 25 °C. The resulting printed scaffolds were kept at room temperature or annealed at 50 °C for 2 days (higher temperatures cause a deformation of the scaffolds), and the crystallinity was measured. Notably, both samples of PLCL (60:40 and 70:30) suffered significant degradation (>60% MW decrease) through the filamenting and 3D-printing process due to the high processing temperatures utilized. These samples were evaluated for degradation in PBS at 37 °C over 28 weeks. The material re-crystallized over a few weeks, accompanied by a considerable increase in brittleness and a decrease in the molecular weight. Understanding these changes to mechanical integrity during hydrolytic degradation is important for the successful use of PLCL polymers in implants [89]. Structures with a higher tortuosity and therefore lower hydraulic permeability result in the accumulation of degradation products inside them, affecting the mechanical stability and disintegration time of the resulting 3D-printed implants.

Work with PLCL has not only been limited to fusion-based printing processes. Chausse et al. [91] prepared radiopaque stents based on PLCL (95:5 PLA/PCL, with a molecular weight of 700 kDa) using a modified PAM printing process. The polymer was dissolved in chloroform, and the rheological properties of the solutions were measured. The solutions exhibited shear thinning, with a viscosity range of 30–400 Pa∙s at apparent shear rates within the range of 10–800 s^−1^. Radiopaque stents with different geometries and thicknesses were successfully printed. They were subjected to mechanical testing, had good mechanical robustness, and most of them withstood the pressure of 16 atmospheres, indicating that they could withstand pressure in blood vessels. The characteristics of the stents depended on the type of radiopaque agent used (iodine, triiodobenzoic acid (TIBA), or barium sulfate (BaSO_4_)), where through microcomputed tomography, the stents’ radiopacity was assessed, showing higher X-ray attenuation values with TIBA and BaSO_4_, maintaining their radiopacity after 3 months upon incubation with PBS at 37 °C. The resistance to compression of the stents printed with a nozzle diameter of 200 μm as opposed to 250 μm was lower, but it increased when a radiopaque agent was added. Except for PLLA10TIBA, where there was a decrease of 30%, no notable change in mechanical characteristics was seen after 3 months of incubation in the remaining samples. Shi et al. [175] synthesized and produced monofilaments of PLLA, PLCL 95/5, and PLGC 80/15/5 in order to be able to generate a comparative study of the accelerated in vitro degradation behavior of the three different compositions of aliphatic polyester monofilaments. The accelerated study was conducted over a range of degradation times from 1 to 21 days at 60 °C, with a post-degradation analysis of filaments via SEM, GPC, DSC, XRD, and tensile testing. The mass loss, T_g_, and morphological integrity of the PLLA monofilament mostly remained intact, according to the degradation data, and partial degradation with a slight increase in crystallinity showed up in the amorphous region. Meanwhile, the PLCL 95/5 monofilament showed a noticeable decrease in mass and T_m_ after 14 days, in addition to an increase in crystallinity, suggesting that most of the amorphous region had been degraded over the course of the accelerated study. The PLGC 80/15/5 monofilament appeared to have the quickest rate of degradation, with significant mass loss and a drop in T_g_. The amorphous zone degraded rapidly in the early stage due to its high water absorbability and low structural uniformity, whereas the first-formed crystalline region degraded slowly, as demonstrated by the shift in crystallinity, and fractured after 3 days. The PLCL 95/5 monofilament degraded 2.5 times faster than the pure PLLA, while the PLGC 80/15/5 monofilament degraded 7.5 times faster than the pure PLLA, as shown by the accelerated effects estimated using the first-order kinetic model.

## 4. Summary

Polymers, as key materials in 3D-printing applications, present a complex landscape due to their diverse structures and properties. This review navigated through the relevant considerations in polymer selection, elucidating the intricate balance required for optimal printability and desired product performance. Examples were provided to understand how the inherent properties of polymers influence the 3D-printing process and how formulators have been selecting polymers for sufficient printability and required product performance in their AM applications. It was highlighted how some polymers or combinations of polymeric systems help to improve the printability of a drug product, while the use of other polymers may only sometimes result in the desired process or product performance. As the pharmaceutical sciences increasingly embrace AM technologies, this review contributes essential insights into the pivotal role of polymers in realizing the full potential of 3D printing for drug-product or medical-device manufacturing.

## Figures and Tables

**Table 1:** Commonly used polymers for pharmaceutical AM applications. DoP = drop-on-powder printing; DoD = drop-on-demand printing; SLS = selective laser sintering; FDM = fused deposition modeling; PAM = pressure-assisted micro-syringe.

Polymer (Alphabetical Order)	Additive Manufacturing Technology				
	Binder Jetting	Material Jetting	Powder-Bed Fusion	Material Extrusion	
	DoP	DoD	SLS	FDM	PAM
Carboxymethylcellulose sodium (Na-CMC)					[28,29,30]
Copovidone (PVP/VA)	[31,32]		[11,33,34,35,36,37]	[9,38,39,40,41,42,43,44,45,46]	
Ethyl cellulose (EC)	[47,48,49]		[50,51]	[38,39,52,53,54,55,56]	
Hydroxyethyl cellulose (HEC)				[57,58]	[59,60,61]
Hydroxypropyl cellulose (HPC)	[24]			[39,40,43,46,52,53,55,57,62,63,64,65]	[66,67]
Hypromellose (HPMC)	[47,48,49]		[33,68]	[38,42,52,53,69]	[25,70,71,72,73,74,75,76]
Polyglycolic acid (PGA)				[77,78]	
Polycaprolactone (PCL)	[79]			[77,80,81,82,83]	[84]
Polylactic acid (PLA)			[85]	[77,78]	
Poly(lactic-co-glycolic) acid (PLGA)		[86,87]			[86,88]
Poly(L-lactide-co-ε-caprolactone) (PLCL)		[89]		[90]	[91]
Povidone (PVP)	[47,48,49,92,93,94,95,96,97,98,99,100]	[23]		[41,80,101,102,103]	[28,71,72,73,75,76,104,105,106]

**Table 2:** Surface tension data of 0.1% (*w*/*w*) solutions of different water-soluble cellulose ethers at 22.0 °C using the Wilhelmy plate technique. Data taken from McMullen et al. [112]. Abbreviations in the table: Me = methyl; HP = hydroxypropyl.

Cellulose Ether	Manufacturer	Approximate Molecular Weight (M_W_, Daltons)	Approximate DS/MS	Surface Tension (mN/m)
Hydroxypropyl cellulose	Ashland, Wilmington, DE, USA	80,000–1,150,000	4.0 MS	41.1–41.7
Hydroxyethyl cellulose	Ashland, Wilmington, DE, USA	720,000–1,300,000	2.6 MS	46.4–49.4
Hypromellose	Ashland, Wilmington, DE, USA	400,000–1,200,000	1.5 Me DS 0.3 HP MS	51.1–54.1
Carboxy methylcellulose sodium	Ashland, Wilmington, DE, USA	250,000–725,000	0.7–1.2 DS	70.5–71.2

**Table 3:** Example of the commercially available grades of hydroxypropyl cellulose (HPC) with data from the manufacturer, Ashland.

Viscosity Grade	Weight Average Molecular Weight (Da)	Viscosity (mPa∙s) in Aqueous Solution	Solution Concentration (%)	Grade Used in 3DP Applications (Based on MW)
HF	1,150,000	1500–3000	1	[52]
MF	850,000	4000–6500	2	[46,55,67]
GF	370,000	150–400	2	
JF	140,000	150–400	5	[62]
LF	95,000	75–150	5	[39,53,64]
EF	80,000	300–600	10	[40,52,53,63,66]
ELF	40,000	150–300	10	[24,43,57,62,65]

**Table 4:** Available pharmaceutical grades of ethyl cellulose according to Brady et al. [22] and 3DP publication references.

Grade	Ethoxyl Substitution %	Average Molecular Weight	Viscosity	Solution Concentration (%)	Grade Used in 3DP Applications
N7	48.0–49.5	65,000	6–8	5	[50,51]
N10	48.0–49.5	75,000	8–11	5	[38,39,55,56]
N14	48.0–49.5	120,000	12–16	5	[52,53]
N22	48.0–49.5	140,000	18–24	5	
N50	48.0–49.5	160,000	40–52	5	
N100	48.0–49.5	215,000	80–105	5	
T10	49.6–51.0	75,000	8–11	5	

**Table 5:** Different grades of PVP with their *K*-values, molecular weights and glass transition temperatures (data from [134,141]).

Grade	Nominal *K*-Value	*K*-Value Range ^a^	Calculated Relative Viscosity of 10% (*w*/*w*) Solution (mm²/s) ^b^	Intrinsic Viscosity (dL g^−1^)	M_W_ (Dalton)	M_n_ (Dalton)	T_g_ (°C)
PVP K-12	12	10.2–13.8	1.48–1.8	0.05	2500 ^e^	1300	120
PVP K-17	17	15.3–18.4	1.98–2.41	0.09	10,000 ^c^	2500 ^d^	140
PVP K-25	25	22.5–27	3.23–4.56	0.16	25,000 ^c^	6000 ^d^	160
PVP K-30	30	27–32.4	4.56–7.14	0.22	40,000 ^c^	10,000 ^d^	164
PVP K-90	90	81–97.2	1075.37–7157.85	1.6	1,100,000 ^c^	150,000 ^e^	174

^a^ according to the European Pharmacopeia, ^b^ calculation based on Equation (1), ^c^ light scattering; ^d^ vapor pressure osmometry; ^e^ gel permeation chromatography.

**Table 6:** Common grades of commercially available copovidone with data from [142,143,144,145].

Product Name	Manufacturer	Molecular Weight (M_n_)	T_g_	Particle Size (x_50_)
Plasdone™ S630	Ashland	14.000–18.000	110.69	<100
Plasdone™ S630 ultra	Ashland	20.000	108.72	<100
Kollidon™ VA64	BASF	15.000–20.000	109	71.1
Kollidon™ VA64 fine	BASF	15.000–20.000	109	16.2

**Table 7:** Properties of synthetic aliphatic polyesters with data from [89,90,148,156,157,158,159,160,161].

Polymer	T_g_ (°C)	T_m_ (°C)	Tensile Strength (MPa)	Tensile Modulus (GPa)	Elongation at Break (%)	Degradation Time (Months)
PGA	35–45	>220	60–100	6–7	1.5–20	6–12
PLLA	55–65	>170	45–70	2–4	3–10	>24
PDLLA	45–55	Amorphous	15–30	<2	2–10	12–16
PLGA (50–85% DLA)	40–55	Amorphous	40–55	1–2	2–10	1–6
PCL	−60	60	20–34	0.2–0.35	>700	>24
PLCL (60–90% LLA)	35–54	156–163	23–27	0.44–1.6	300–379	3–12

## Data Availability

The data presented in this study are available in this article.
